# Post-cholecystectomy bile duct injuries: a retrospective cohort study

**DOI:** 10.1186/s12893-023-02301-2

**Published:** 2024-01-03

**Authors:** Mohamed Hossam El-Din Zidan, Mostafa Seif-Eldeen, Abdelhamid A Ghazal, Mustafa Refaie

**Affiliations:** 1 Faculty of Medicine, Alexandria University, Alexandria, Egypt; 2 Alexandria Main University Hospital, Alexandria University, Alexandria, Egypt

**Keywords:** Biliary duct injuries, Cholecystectomy, ATOM classification, Re-laparoscopy, Diagnostic laparoscopy, Strasberg classification, Laparoscopic cholecystectomy, Open cholecystectomy, Bile duct injury, Laparoscopic roux-en Y hepaticojejunostomy, Clavien-Dindo classification

## Abstract

**Background:**

Bile duct injury (BDI) is still a major worrisome complication that is feared by all surgeons undergoing cholecystectomy. The overall incidence of biliary duct injuries falls between 0.2 and 1.3%. BDI classification remains an important method to define the type of injury conducted for investigation and management. Recently, a Consensus has been taken to define BDI using the ATOM classification. Early management brings better results than delayed management. The current perspective in biliary surgery is the laparoscopic role in diagnosing and managing BDI. Diagnostic laparoscopy has been conducted in various entities for diagnostic and therapeutic measures in minor and major BDIs.

**Methods:**

35 cases with iatrogenic BDI following cholecystectomy (after both open and laparoscopic approaches) both happened in or were referred to Alexandria Main University Hospital surgical department from January 2019 till May 2022 and were analyzed retrospectively. Patients were classified according to the ATOM classification. Management options undertaken were mentioned and compared to the timing of diagnosis, and the morbidity and mortality rates (using the Clavien-Dindo classification).

**Results:**

35 patients with BDI after both laparoscopic cholecystectomy (LC) (54.3%), and Open cholecystectomy (OC) (45.7%) (20% were converted and 25.7% were Open from the start) were classified according to ATOM classification. 45.7% were main bile duct injuries (MBDI), and 54.3% were non-main bile duct injuries (NMBDI), where only one case 2.9% was associated with vasculobiliary injury (VBI). 28% (n = 10) of the cases were diagnosed intraoperatively (Ei), 62.9% were diagnosed early postoperatively (Ep), and 8.6% were diagnosed in the late postoperative period (L). LC was associated with 84.2% of the NMBDI, and only 18.8% of the MBDI, compared to OC which was associated with 81.3% of the MBDI, and 15.8% of the NMBDI. By the Clavien-Dindo classification, 68.6% fell into Class IIIb, 20% into Class I, 5.7% into Class V (mortality rate), 2.9% into Class IIIa, and 2.9% into Class IV. The Clavien-Dindo classification and the patient’s injury (type and time of detection) were compared to investigation and management options.

**Conclusion:**

Management options should be defined individually according to the mode of presentation, the timing of detection of injury, and the type of injury. Early detection and management are associated with lower morbidity and mortality. Diagnostic Laparoscopy was associated with lower morbidity and better outcomes. A proper Reporting checklist should be designed to help improve the identification of injury types.

**Supplementary Information:**

The online version contains supplementary material available at 10.1186/s12893-023-02301-2.

## Background

Post-cholecystectomy Bile duct injury (BDI) is associated with morbidity and mortality making it the most feared complication of cholecystectomy [1, 2]. BDI is in direct correlation with surgical experience and knowledge of cholecystectomy. Past studies in the last three decades stated that the incidence of BDI after laparoscopic cholecystectomy (LC) was significantly greater than that after open cholecystectomy (OC) (0.4–0.6% and 0.1–0.2%, respectively). This was corresponding to the emergence of the laparoscopic technique in that era [3, 4]. However, Later studies found a considerable decline in the incidence of BDIs after LC, to around 0.2%, due to the improved surgical laparoscopic experience [5].

Prevention of BDI remains the most important aspect in the application of the surgeon’s learning curve. Prevention of BDI, is through thorough knowledge of the mechanism by which a BDI occurs, understanding the critical view of safety, and a proper selection of patients [6–8]. The World Society of Emergency Surgery (WSES) guidelines in 2020, advocate the implementation of a “Bailout” surgery in cases of obscure anatomy, to prevent BDI [9]. Bailout surgeries include Subtotal Cholecystectomy, or Cholecystostomy insertion followed by interval cholecystectomy [9, 10]. Although Conversion to OC is an option to enhance visualization, there is no sufficient evidence to support the fact that conversion decreases the rate of BDI [9].

The timing of detection of BDI remains the most important variable in managing BDI, which significantly affects the outcome of the patients, regarding morbidity, general well-being, and mortality. Many studies were formulated to interpret the timing of injury detection in correspondence with the mortality rates, these data concluded that the time of BDI detection is important, but that there are very few cases of BDIs recognized intraoperatively, despite the wide ranges (25–92%) reported in the literature [11, 12].

To reduce major morbidity, it is crucial to identify BDI as soon as possible in patients who experience an unusual course after cholecystectomy. As a result, imaging techniques like ultrasound and computed tomography (CT) are very helpful during the initial assessment of a patient with a BDI. Intraoperative findings, clinical evidence, Diagnostic Laparoscopy (DL), and post-operative imaging, including Endoscopic Retrograde Cholangiopancreatography (ERCP), Magnetic Resonance Cholangiopancreatography (MRCP), Computed Tomography (CT), and Percutaneous Transhepatic Cholangiography (PTC) can all be used to identify BDI [9, 13].

**Fig. 1:**
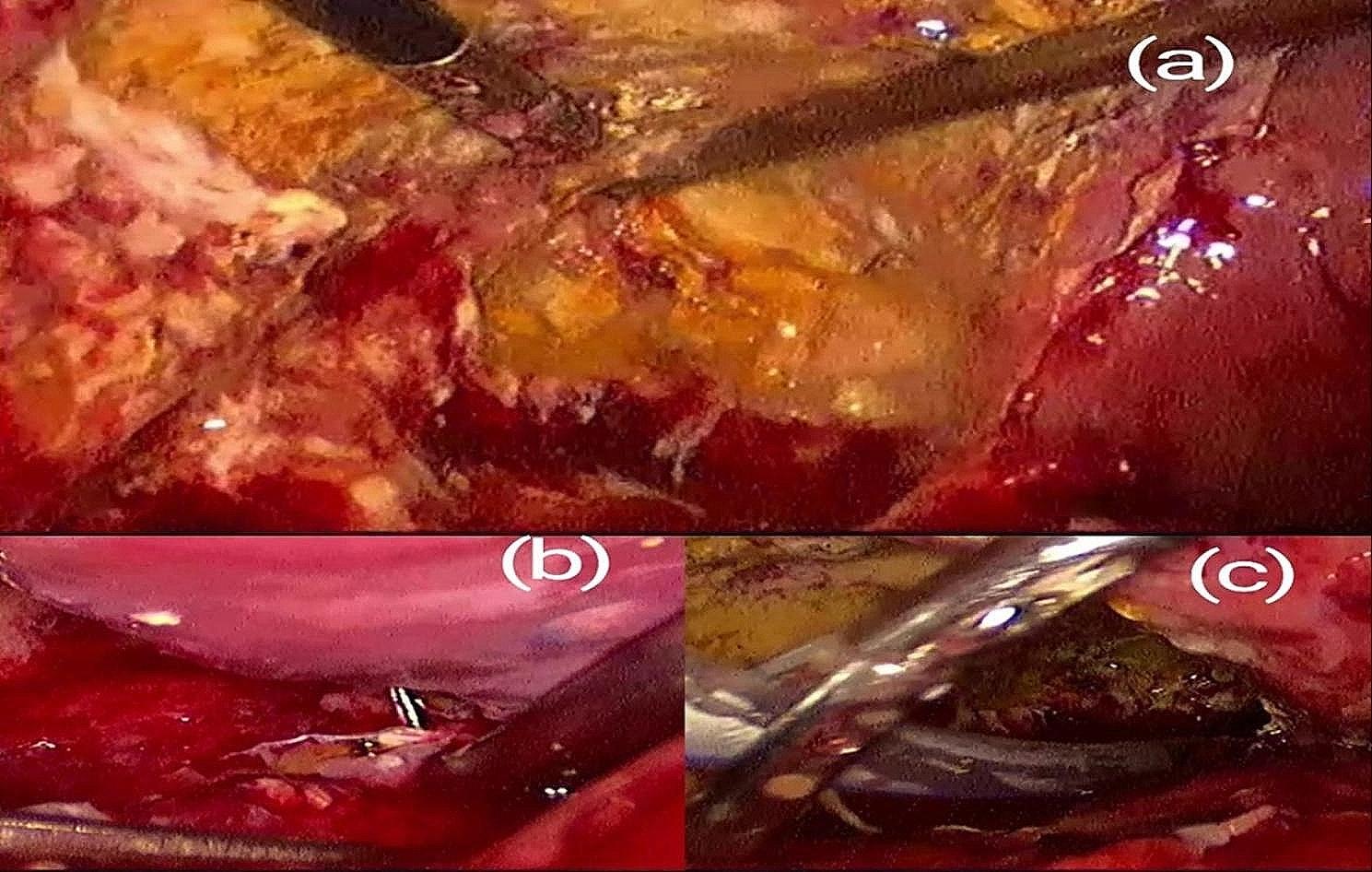
Relaparoscopy in a minor BDI. (**a**) lavage and exploration of the gallbladder bed revealed residual biloma that had been aspirated. (**b**) clipping of a small duct with a suspected biliary leak at the Subvesical region. (**c**) drain insertion

Although re-laparoscopy is not yet advocated by the current guidelines, this modality is helpful in not only assessing and identifying the injury but also in managing BDIs. Re-laparoscopy can rule out duodenal injuries, treat minor BDIs, and extensively irrigate and drain the abdomen [14–16] (Fig. 1). This method can be used to treat the injury conservatively or to stabilize the patient while the definitive repair is planned. Early post-operative referred patients with suspected BDI in the first 72 h, are good candidates to relaparoscopy. Surgeons can choose whether to proceed with immediate repair or delay the repair of the injury, depending on the location and extent of the injury, the patient’s overall stability, and local expertise [16].

Nonetheless, classifying BDI remains an important aspect of the management of BDI. Surgeons have long struggled to define and categorize BDIs. Various approaches have been employed throughout the surgical literature, but no consensus was reached until the WESES guidelines of 2020, which advocated the use of the ATOM classification as a comprehensive classification [9, 17].

Although Surgical reconstruction of the biliary continuity is the mainstay of treatment, management options should be customized based on variables such as the timing of BDI diagnosis, and the presence of sepsis, or coagulopathy. Management of BDI is discussed according to the type of injury and the time of detection of BDI.

## Patients and methods

A power analysis was conducted to determine the sample size of 35 patients in a retrospective cohort study that analyzed post-cholecystectomy biliary duct injuries. The analysis showed a probability of 82% that the statistical tests used are significant. The study examined patients who either experienced or were referred to Alexandria Main University Hospital surgical department, Egypt from January 2019 till May 2022. The patients had varying presentations and timings of post-cholecystectomy biliary tract injuries. The study involved a thorough examination of the patient’s clinical data, which was collected using a checklist designed by the department in 2020. Some data were missing in older databases, but most of it was recovered through phone calls to the patients.

Post-operative methods for diagnosing BDI were both objective and subjective presentations followed by a thorough workup. Subjective presentations included abdominal pain, distension, nausea, fever, and malaise. Objective presentations included evidence of bile leakage from a post-operative drain, obstructive jaundice, sepsis, septic shock, and signs of biliary peritonitis. Ultrasound was done routinely in all cases with a suspected injury. However, highly suspected cases underwent a more rigorous workup, including MRCP, PTC, or ERCP. “Drain tube cholangiography” was also an option in patients with BDI diagnosed intra-operatively by a non-biliary surgeon who drained the biliary system with a tube (e.g., T-tube) and referred the patient to our hospital. CT scan was not done routinely, except in cases with prolonged control, to assess intraperitoneal collections and biloma or in cases that developed pancreatitis following ERCP. DL was chosen in certain cases to both diagnose the underlying disease and as an imperative therapeutic agent.

An intra-operative diagnosis of the Main Biliary Duct (MBD) injury, Strasberg E1-5, was managed by both conventional and laparoscopic bilioenteric anastomosis. Non-Main Biliary Duct (NMBD) injury, Strasberg A-D, was managed intraoperatively by either ligation of the leaking structure, or direct repair.

Managing options of BDI depended on the anatomic level, type, extent of injury, timing of detection, and the presence or absence of the vascular-biliary system. Patients were classified according to the Strasberg classification; However, in the recent year 2022, our department has changed its way of looking into biliary injuries to use the ATOM classification to define all biliary injuries. Thereby, Previous records in this study were re-classified by the ATOM classification.

Reconstructive surgery is the most definitive treatment for bile duct injury cases; however, not all BDIs need reconstructive surgery. Bilio-enteric bypass surgery was decided to treat intra-operatively detected BDI immediately and electively 6 weeks after drainage of both early postoperative and late diagnosed BDIs. Open and laparoscopic approaches have been done on different occasions in our facility. Our preferred method of bilio-enteric anastomosis was Roux-en-Y hepaticojejunostomy. Laparoscopic Roux-en-Y hepaticojejunostomy was fashioned one time in this study.

Post-bilioenteric bypass leakage was either managed by pharmacological treatment and follow-up, or drainage, and interventional radiology. The clinical outcomes after bilioenteric anastomosis were classified according to the Terblanche classification [18].

we reviewed our cases and classified them according to the Clavien-Dindo Classification [19], to assess and evaluate management modalities and their effect on patients with morbidity and their general well-being. The classification and comparison were based on the type of therapy required to treat the complication. The rationale for preserving this approach was to eliminate subjective interpretation of serious adverse events and any tendency to down-grade complications. To avoid imprecision in reporting complications, we avoided using qualitative terms, such as “major” and “minor” complications. We retained these terms to only classify BDIs and not their sequel.

### Statistical analysis

power analysis was performed using Jamovi version 2.3 to determine the required sample size. Data was analyzed using IBM SPSS software package version 20.0. **(**Armonk, NY: IBM Corp**)**. The chi-square test was used to compare the managing options between groups, and the difference was considered statistically significant at *P* ≤ 0.05.

### Utilization of the literature

In our research, we employed various classification systems to accurately define BDI, stratify the complications, and classify the level of stricture. Supplementary file 2 (Appendix 2), discusses all the classification systems we utilized and includes references to the original works. Additionally, we have included tables to clearly illustrate these classification systems, ensuring that even readers unfamiliar with them can comprehend the data we presented. The classification systems utilized from the literature were the Strasberg classification [20], The ATOM classification [17], The Clavien-Dindo Classification [19], and the Terblanche classification [18]. The uses of these classifications and their importance are all referenced both directly in supplementary file 2, and indirectly in this article.

## Results

### Patient demographics and pre-operative data

A total of 35 patients with bile duct injuries were identified and their clinical data was reviewed. Female to male ratio was 1.97:1, and the median age was 37.97 (range, 19–57) (Table 1). patients were identified according to the type of operation, operative time, and method of cholecystectomy (Table 2). 13 cases (37.1%) of bile duct injuries occurred at our hospital Alexandria Main University Hospital, and 22 cases (62.9%) referred from secondary hospitals with suspicion of BDI, or a definitive diagnosis (supplementary File 1, Table 1).

Indications of cholecystitis were identified retrospectively, where 65.7% had dyspeptic symptoms or a history of biliary colic, 20% had acute cholecystitis, and 25.8% had other modes of presentation.

MBD injury was identified in 16 cases, and NMBDI was identified in 19 cases. LC was responsible for only 18.8% of MBD injuries, compared to OC and converted cases which were responsible for 43.8% and 37.5% of MBD, respectively. However, the rate of NMBD injuries was much higher in LC (83%) compared to OC (10.5%) and converted cases (5.3%) (Table 2).

The conversion was recorded in 20%, where justifications of conversions were recorded, including obscured anatomy in 28.6%, previous multiple abdominal surgeries in 28.6%, previous cholecystostomy drainage in 14.3%, failure to control bleeding in 14.3%, and stone impaction at the neck in 14.3%.

**Table 1:** Clinical characteristics of 35 patients with bile duct injuries

Pre-operative clinical characteristics		
Age (median, range), years.	38	19–57
**Sex (n)** Male Female	**N** 12 23	**%** 34.3 65.7
**Type of operation**	**N**	**%**
Elective	23	65.7
Urgent	12	34.3
**Operative Time (n = 35)**	**N**	**%**
Unrecorded	3	8.6
<120	11	31.4
>120	21	60
**Method of cholecystectomy**	**N**	**%**
Laparoscopic (LC)	19	54.3
Open (OC)	9	25.7
Converted	7	20

**Table 2:** Method of cholecystectomy concerning the type of biliary injury

	MBD/NMBD injury					
**Method of cholecystectomy**	**MBD injury** **(n = 16)**		**NMBD injury** **(n = 19)**		χ^2^	^*P*−value^
	**No.**	**%**	**No.**	**%**	15.017	0.001
Laparoscopic Cholecystectomy (LC)	3	18.8	16	84.2		
OC from the start	7	43.8	2	10.5		
LC converted to OC	6	37.5	1	5.3		

### Classification of BDI

Before 2022, all patients were recorded with the Strasberg classification (Supplementary File 1, Table 2); however, important data that helps plan management, including the presence/absence of VBI, the timing of detection, and the extent of injury were missing from this classification. Thus we retrospectively adopted the ATOM classification, and all missing data was retrieved through phone calls to patients, original referred-from centers and surgeons, and radiological data.

### Patients according to ATOM classification

Minor bile duct injuries (NMBD) were more detailed in the EAES classification (ATOM) (Table 3). We have identified 1 case (2.9%) with cystohepatic accessory duct injury (also called aberrant cystic duct), 8 cases (22.8%) were classified as the accessory duct of Luschka or aberrant Subvesical bile ducts, 7 cases (20%) with a leak from the cystic stump due to a slipped clip, 1 case (2.9%) with an aberrant right hepatic duct, and 2 cases (5.7%) with lateral injury to the biliary tree without tissue loss. The type and extent of injuries were classified into partial division (37.1%), partial occlusion (14.3%), complete division (40.0%), and complete occlusion (8.5%). Only one case was reported with Vasculo-biliary injury (VBI) and hepatic abscess formation (2.9%).

Timing of detection was classified into three main groups: Intraoperative identification (Ei) (28.6%), early postoperative identification (Ep) (identified up to 3 weeks post-cholecystectomy) (62.9%), and late postoperative identification (L) (identified more than 3 weeks post-cholecystectomy) (8.6%). Ei was subdivided by the method of identification by either intraoperative cholangiography (2.9%), identification of duct injury by vision (devisu) (5.7%), or evidence of bile leak (bile leak) (20%). Ep was subdivided into cases identified within the first 72 h (25.7%), and cases identified in the window between 72 h and 3 weeks (74.3%).

The mechanism of injury was defined by the ATOM classification into either mechanical injury (Me) or energy-driven (ED); However, we found that in 54.3% of cases, the mechanism of injury could not be identified. Of the remaining cases, 37.1% were due to mechanical injury and 8.6% were energy-driven (Table 3). The lack of sufficient reported evidence in referred cases contributed to the high rate of unknown mechanisms of injury.

**Table 3:** Classification of bile duct injuries in 35 patients according to ATOM [17]

ATOM Classification (n = 35)	No.	%
Anatomical Level		
MBD (Major)	16	45.7
• MBD 1	6	17.1
• MBD 2	6	17.1
• MBD 3	1	2.9
• MBD 4	1	2.9
• MBD 5	2	5.7
• MBD 6	0	0.0
**NMBD (Minor)**	**19**	**54.3**
• Cystohepatic accessory duct	1	2.9
• The duct of Luschka (hepatico-cholecystic bile duct) / Aberrant Subvesical bile duct	8	22.8
• A leak from the cystic stump (slipped cystic duct clip)	7	20.0
• Aberrant right hepatic duct	1	2.9
• Lateral injury to CHD	2	5.7
**Type and extent of the injury**		
Partial division	13	37.1
Complete division	14	40.0
Partial occlusion	5	14.3
Complete occlusion	3	8.5
**Vasculobiliary injury**		
No	34	97.1
Right hepatic artery	1	2.9
**Time of detection**		
**Intraoperative (Ei)**	**10**	**28.6**
• Devisu	2	5.7
• bile leak	7	20.0
• IOC	1	2.9
**Early Postoperative (Ep)**	**22**	**62.9**
• < 72 h.	9	25.7
• > 72 h. <3 weeks	13	74.3
**Late (L)**	3	8.6
**Mechanism of damage**		
Unknown	19	54.3
Mechanical (Me)	13	37.1
Energy-driven (ED)	3	8.6

### Management of intra-operatively detected BDI

**Fig. 2:**
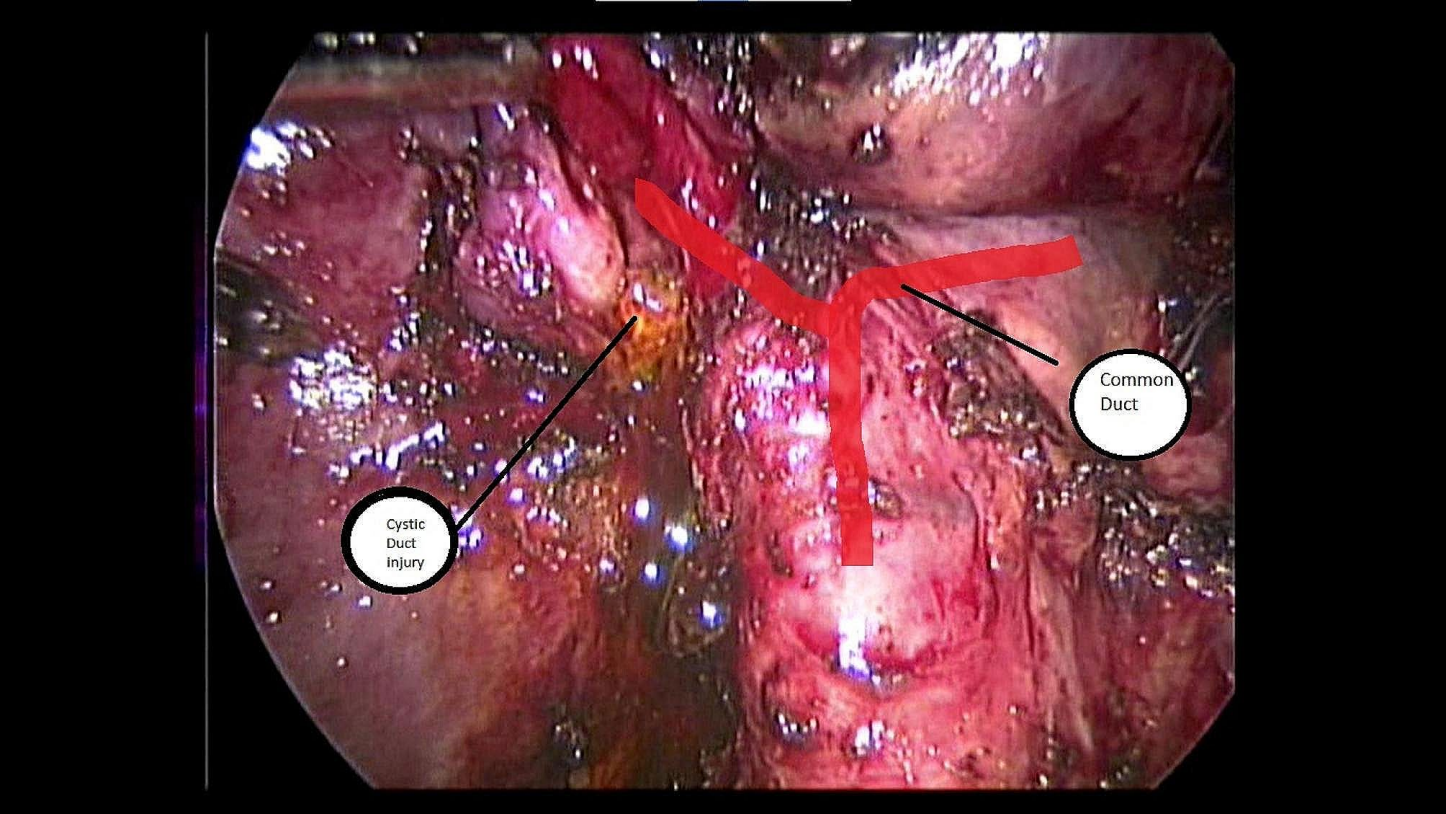
Intraoperatively detected bile leak – Strasberg A

BDI was diagnosed intraoperatively in 10 cases (28.6%); of which, 4 cases (11.4%) were MBD injuries, and 6 cases (17.14%) were NMBD injuries (Fig. 2). Intraoperative detection was done by Intraoperative cholangiogram (IOC) in one case, after evidence of bile leak in 7 cases, and after noticing a defect (devisu) in 2 cases.

**Fig. 3:**
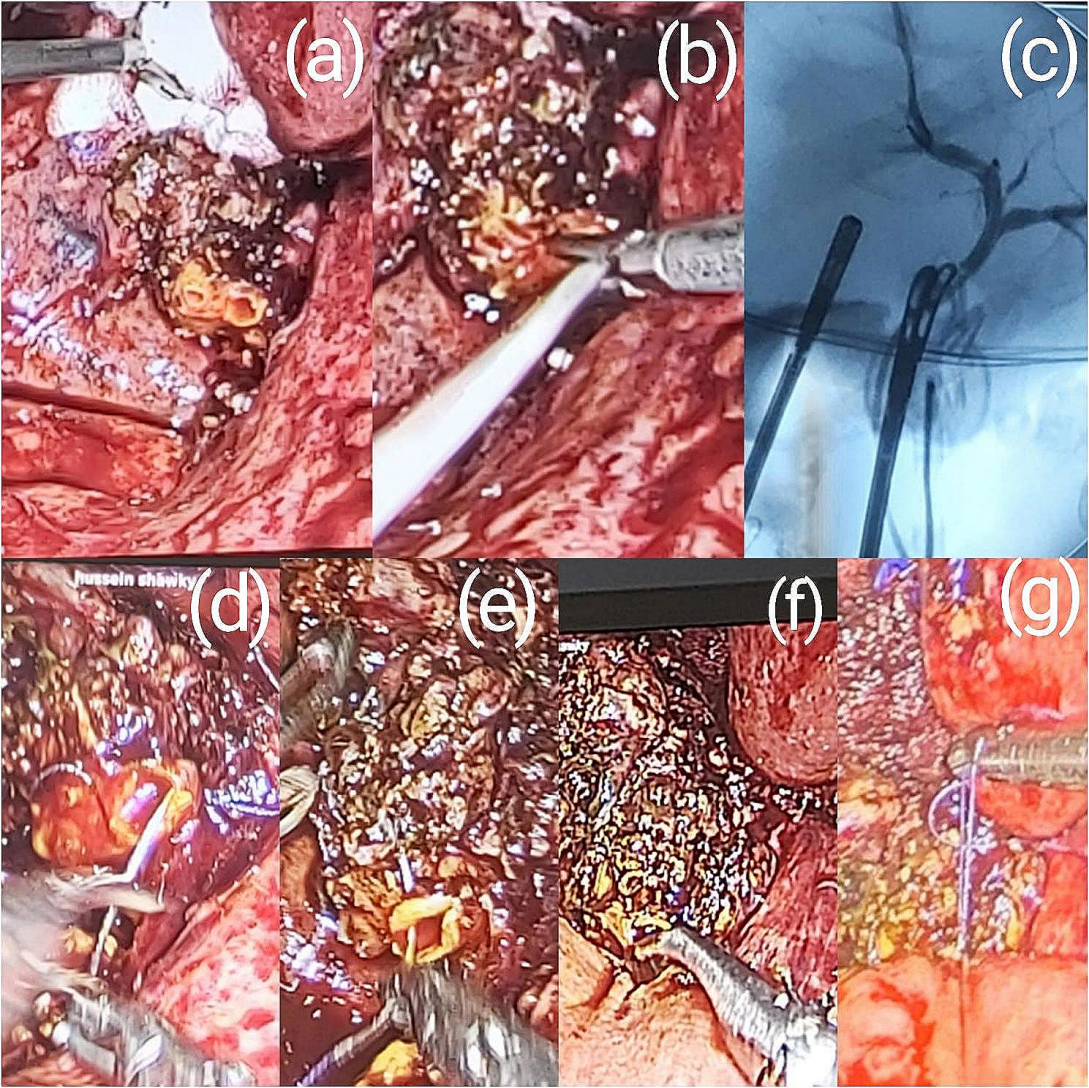
An intraoperatively diagnosed BDI managed by laparoscopic Roux-en-Y hepaticojejunostomy. (**a**) shows the transected biliary duct, (**b**) A catheter inserted in the bile ducts for performing an IOC. (**c**) shows an IOC image taken with good flow to both lobes. (**d**), (**e**), and (**f**) show sutures being taken in the bile duct, preparing for hepaticojejunostomy. (**g**) primary hepaticojejunostomy performed, jejunal loop seen in the image adhering to the previously mentioned bile duct

There were two cases of intraoperatively diagnosed major BDI’s that were reconstructed in the same setting by two different experienced hepatobiliary surgeons (who were also responsible for the BDI). One of the two definitive reconstructive surgeries was done laparoscopically (Fig. 3) and the other was conventional Roux-en-Y hepaticojejunostomy. The laparoscopically performed Roux-en-Y hepaticojejunostomy (Fig. 3) had good results with no leakage and needed no further intervention in follow-up; However, the conventional hepaticojejunostomy was followed by a biliary leak that was managed by an external PTD. At follow up the patient was well for 3 weeks and removed the PTD. There were 6 cases of the intraoperatively diagnosed duct of Luschka (hepatico-cholecystic bile duct) and aberrant Subvesical bile duct injuries, that were clipped or ligated using a 4/0 Vicryl or PDS suture, all six cases did well during the postoperative period and needed no further management.

**Fig. 4:**
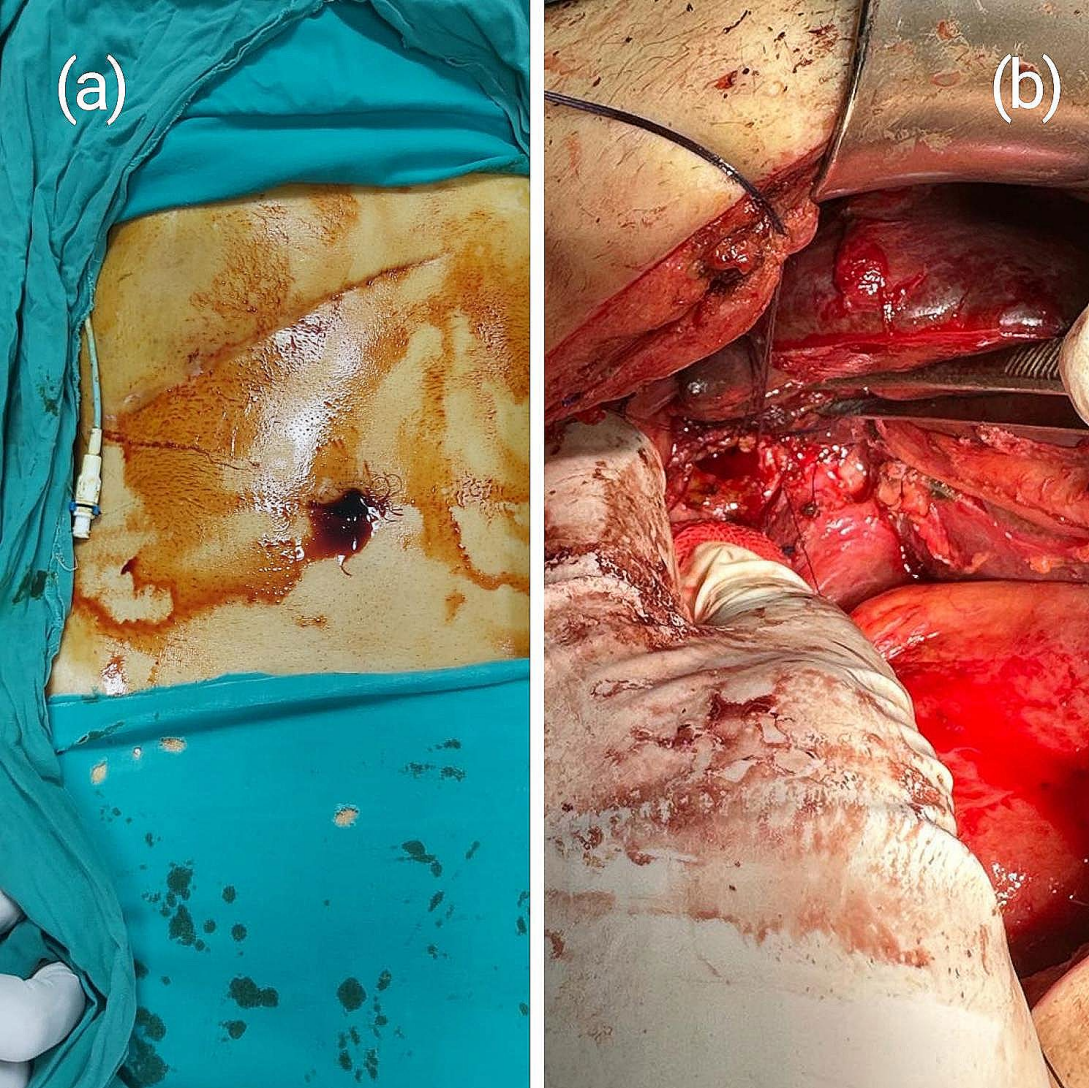
Intraoperatively diagnosed patient with BDI that was triaged by ligation of the BD. PTBD was then inserted to drain the BD, and a reconstructive bilioenteric bypass was undergone after 10 weeks. (**a**) shows a PTD on the right side of the patient which was inserted weeks earlier and the previous scar of the operation. (**b**) shows a CHD after dissection and is ready for hepaticojejunostomy

Triaging surgeries are surgeries done in cases where there are no experienced hepatobiliary surgeons. Two intraoperatively diagnosed MBD injuries were triaged: one by a T-tube inserted at the injury site to facilitate tube cholangiography and further repair, and another case was triaged by ligature of the common hepatic duct without marking of the biliary duct, which prolonged the postoperative management and entailed further biliary stenting with a PTD. ERCP with sphincterotomy was conducted on the T-tube triaged patient, to decrease the intra-biliary pressure, and facilitate biliary drainage. The two triaged cases were eligible for delayed hepaticojejunostomy 6 weeks later as a definitive reconstructive procedure (Fig. 4).

### Management of post-operatively detected BDI

**Fig. 5:**
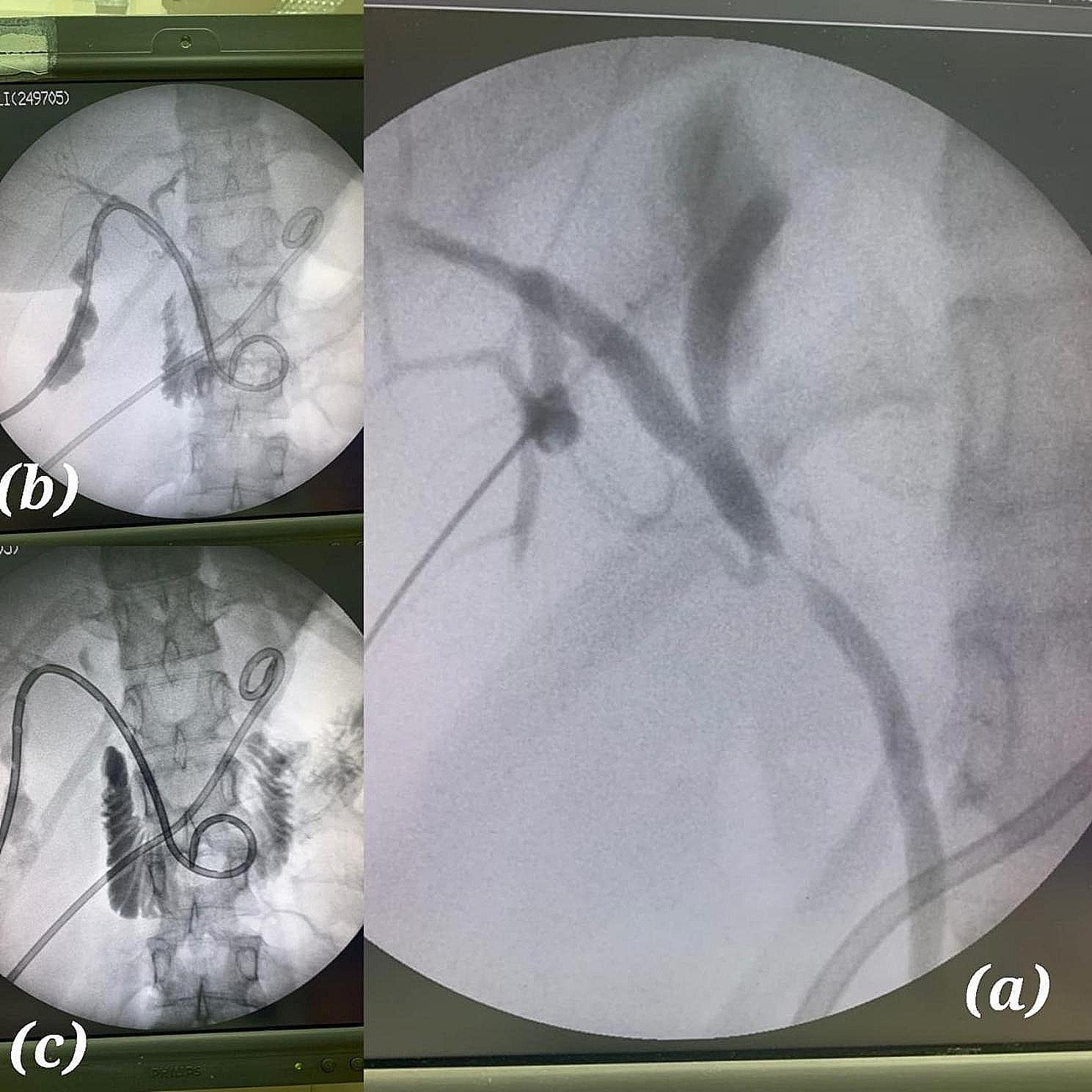
A case of delayed management of BDI. (**a**) shows a Chiba needle inserted for dye insertion and PTC showing a lateral leak and stricture in the CHD. (**b**) Percutaneous transhepatic drain (PTD) was inserted external to internal to reach the duodenum and showed no extravasation of dye with positive contrast at the duodenum. (**c**) Followup PTC 10 days after the insertion of the PTD showing no extravasation of dye. Note a Pigtail in all 3 images transecting the view to a previously mentioned collection

postoperative diagnosis of a patient with a BDI was analyzed in 25 patients, 22 patients were diagnosed in the early postoperative period, and 3 cases were diagnosed in the late postoperative period (Fig. 5). Subjectively, these patients reported nausea, fever, and malaise in 60%, and abdominal pain in 64%. Objectively, they have shown evidence of obstructive jaundice in 20%, sepsis in 8%, and documented bile leak in 72%.

Post-operative investigations were undertaken on all clinically suspected patients. Non-invasive investigations were the first investigations conducted in most cases. Abdominal ultrasonography was the initial investigation of choice, in 88%, Magnetic resonance cholangiopancreatography (MRCP) in 72%, and Computed tomography with intravenous contrast (CT-IV) in 60%. (Supplementary File 1, Table 3)

Invasive investigations were chosen with caution, as they further affect the morbidity and outcome of the patients. The choice of intervention was implemented to not only diagnose, but also to manage the cases; therefore, it is important to carefully choose the intervention at hand to both plan for biliary drainage, and abdominal drainage.

**Fig. 6:**
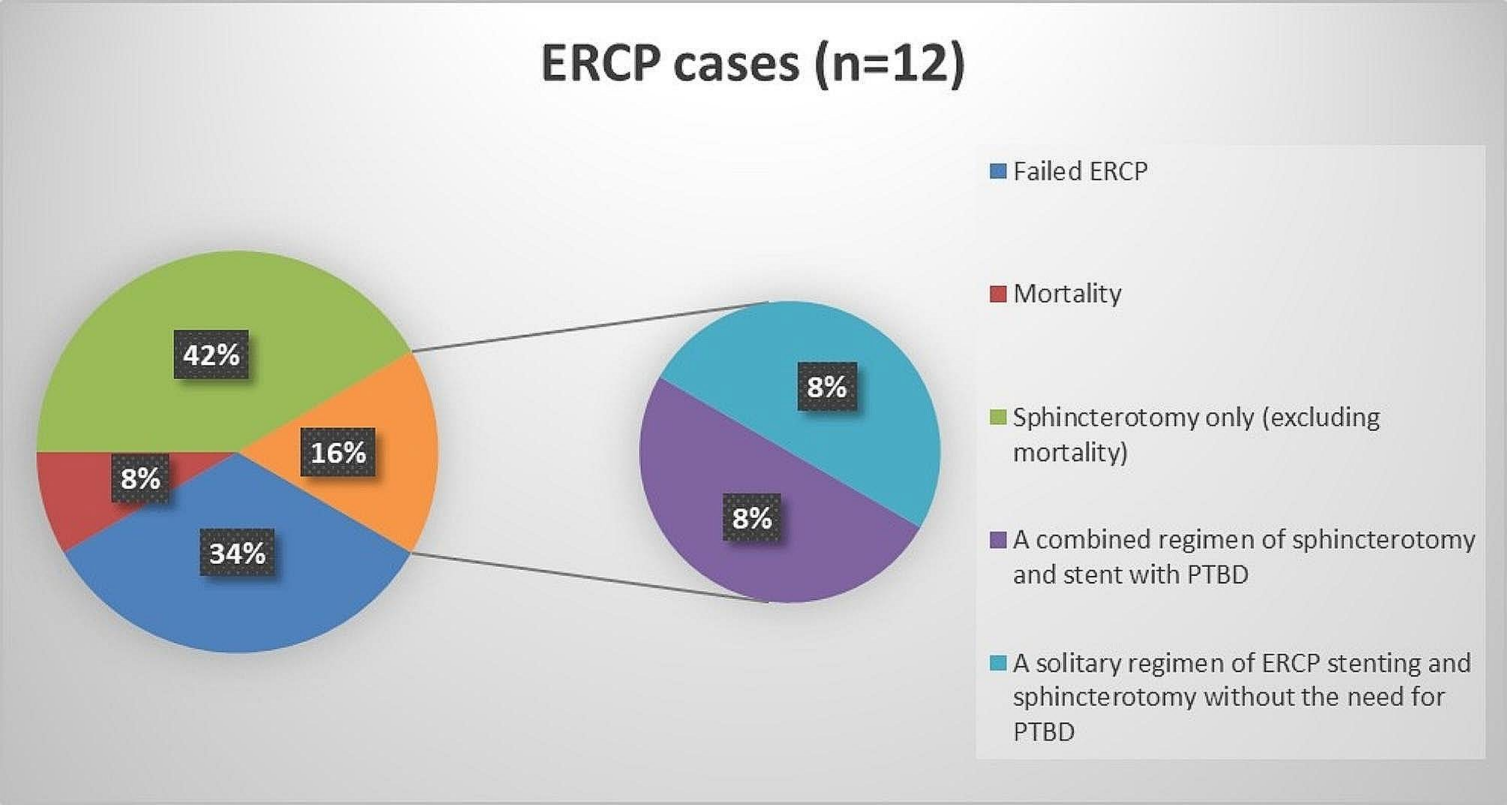
The Pie chart shows all cases of ERCP n = 12 conducted in our study: (**a**) Failed ERCP 33.33 (**b**) Mortality caused by ERCP and sphincterotomy (8.33%) (**c**) Sphincterotomy only, after excluding the mortality case (41.67%) (**d**) Sphincterotomy and stent (16.67%). The right small pie chart demonstrates the stented cases where (d1) a combined regimen of ERCP stenting and PTBD, and (d2) a solitary regimen of ERCP sphincterotomy and stenting alone

Early postoperative (Ei) diagnosed bile duct injuries are further classified into two subgroups according to the urgency of intervention needed. MBD injury Cases diagnosed in the first 72 h are liable for bilioenteric anastomosis if referred promptly to a tertiary hospital with hepatobiliary expertise, NMBD injury patients diagnosed in the same subgroup are also amenable to definitive repair. 9 patients were diagnosed in this subgroup (< 72 h), all of which were treated with re-laparoscopy. ERCP was conducted on 3 cases as a supplementary modality of treatment in the same setting of anesthesia with re-laparoscopy (hybrid technique). Out of the three cases of ERCP, Sphincterotomy was done in 2 cases to decrease the intra-biliary pressure and improve healing; Stent insertion and sphincterotomy were done in one case to bypass the site of injury (Fig. 6).

**Fig. 7:**
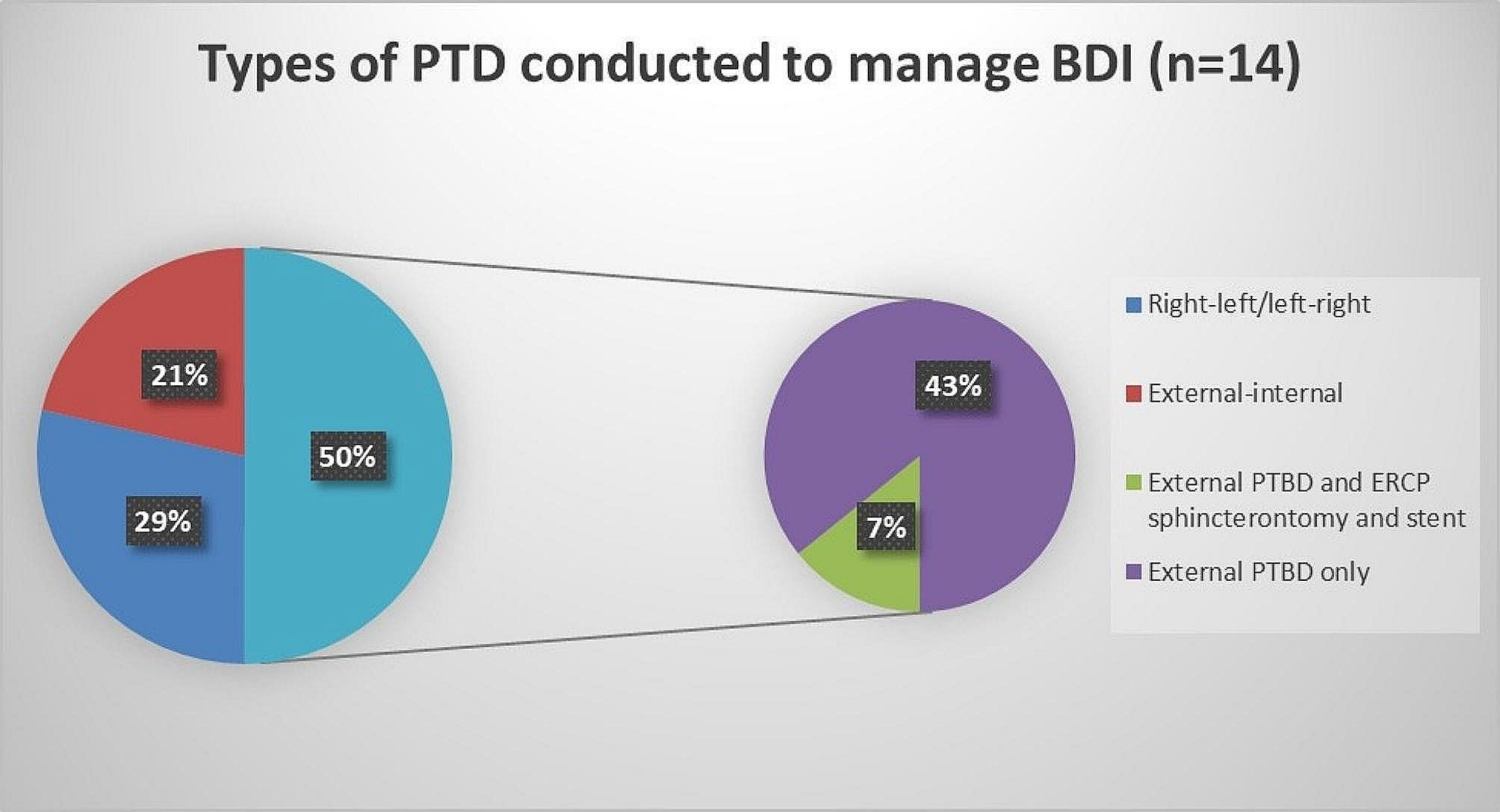
Pie chart shows types of PTD conducted in the study to manage BDI (n = 14)

13 patients were diagnosed after 72 h from the primary operation. Delayed hepaticojejunostomy was the definitive treatment for 11 patients in this subgroup, and relaparoscopy was definitive in 7.7% (n = 1). However, one 46-year-old case (7.7%) died in this subgroup due to severe post-ERCP pancreatitis. Percutaneous transhepatic biliary drainage (PTBD) was the main modality of investigation, and biliary drainage in 11 cases of this subgroup (84.6%), all of which were later amenable for bilioenteric anastomosis (Fig. 7). ERCP was done in 23.1% of the cases, with stent insertion undergone in 7.7% and sphincterotomy only in 15.4% (Fig. 6). Pigtail insertion was inserted in 53.8% of the cases in this timeframe for abdominal drainage of bilomas.

There were 3 cases (8.6%) diagnosed in the late postoperative period. Biliary drainage and abdominal drainage were needed in all 3 cases in this subgroup. 2 cases needed abdominal drainage with pigtail insertion and biliary drainage with PTBD, and one case (33.3%) was diagnosed with septic shock and was surgically explored to both drain the abdomen and insert a biliary stent. Surgically drained biliary stent was used to both decrease biliary spillage and enhance “tube cholangiography,” for follow-up radiology before reconstruction. 66.7% of the patients in this timeframe needed pigtail insertion for abdominal drainage. Delayed hepaticojejunostomy was the definitive treatment for 66.7% of this subgroup. Mortality was recorded in one 22-year-old case in this subgroup, due to delayed intervention, which led to electrolyte imbalance, and death, even after pigtail insertion and PTBD. ERCP with sphincterotomy was used to ensure low pressure in the biliary tract in one case.

ERCP was done in 12 cases, 4 of which (33.3%; n = 12) had failed, 1 case (8.3%; n = 12) had died after ERCP with sphincterotomy due to post-ERCP pancreatitis, 5 cases underwent sphincterotomy only (41.7%; n = 12), and 2 cases did sphincterotomy with stent insertion (16.7%; n = 12).

All the management options undertaken in relation to the time of detection are summarized in (Table 4).

**Table 4:** Management procedures in 35 patients with bile duct injuries following cholecystectomies with respect to the timing of detection

Management options undertaken	Time of detection								χ^2^	^MC^p
	Early intraoperative (Ei) (n = 10)		Early Postoperative (Ep) (n = 22)				Late (L) (n = 3)			
	No.	%	-<72 h. (n = 9)		->72 h. - <3 weeks (n = 13)		No.	%		
			No.	%	No.	%				
PTBD	2	20.0	0	0.0	11	84.6	2	66.7	19.378^*^	< 0.001^*^
Successful ERCP	**1**	**10.0**	**3**	**33.3**	**3**	**23.1**	**1**	**33.3**	2.057	0.531
Sphincterotomy Only	1	10.0	2	22.2	2	15.4	1	33.3	1.650	0.725
Sphincterotomy + Stent	0	0.0	1	11.1	1	7.7	0	0.0	1.894	0.780
Surgical Exploration	0	0.0	0	0.0	0	0.0	1	33.3	5.545	0.087
Re-Laparoscopy	0	0.0	9	100	1	7.7	0	0.0	27.572^*^	< 0.001^*^
Primary bilioenteric anastomosis	2	20.0	0	0.0	0	0.0	0	0.0	3.805	0.305
Delayed Hepaticojejunostomy	2	20.0	0	0.0	11	84.6	2	66.7	19.378^*^	^MC^p<0.001^*^
Ligature/clipping of Duct of Luschka	6	60.0	0	0.0	0	0.0	0	0.0	13.639^*^	^MC^p<0.001^*^
T-tube Insertion	1	10.0	0	0.0	0	0.0	0	0.0	3.137	0.629
Ligature of a main duct as a method of triaging *	1	10.0	0	0.0	0	0.0	0	0.0	3.137	0.626
Pigtail drainage	1	10.0	0	0.0	7	53.8	2	66.7	11.004^*^	^MC^p=0.005^*^

(PTBD) Percutaneous Transhepatic Biliary Drainage; (ERCP) Endoscopic Retrograde cholangiopancreatography; (χ^2^): Chi-square test; (MC) Monte Carlo; (p) *p*-value; *ligature of a main duct during intra-operative diagnosis is not recommended by current literature [21]

The severity of the injury was classified according to Clavien-Dindo. Of the injuries, 68.6% were classified as IIIb, 20% as I, 5.7% as V (mortality rate), 2.9% as IIIa, and 2.9% as IV. The follow-up of patients surviving the post-operative period was 6 months. Both cases classified as V were NMBD injuries. We have compared the Clavien-Dindo classification to the level of injury (MBDI/NMBDI) (Supplementary file 1, Table 4) and the type of management undertaken. (Supplementary File 1 Table 5).

Hepaticojejunostomy was done in 17 cases (48.6%) of all cases diagnosed with BDI. Complications related to hepaticojejunostomy were wound infection in 11.4% (4/17), and leakage in 20% (7/17). The clinical outcomes of bilioenteric reconstruction cases (n = 17) were classified according to Terblanche et al. [18] for at least 6 months. Out of these cases, 88.2% (15/7) showed no biliary symptoms (Grade 1), while the remaining 11.8% (2/17) were classified as Grade 3, as they had undergone Percutaneous transhepatic stenting and diversion. Both cases healed spontaneously, the stent was removed 6 weeks later, and underwent a serial follow-up MRCP at 3 months, 4 months, and 6 months from the date of bilio-enteric anastomosis, with no recorded stricture up until the date of this article. Both the mortality and morbidity sequel are summarized in Supplementary File 1, Table 6.

## Discussion

Biliary duct injury remains the most perilous complication of cholecystectomies, despite the increasing knowledge of gallbladder surgeries. Since 1996, LC has sought its trending course to become the gold standard technique. On the contrary, many reviews depicted a lower rate of BDI after OC (0.1–0.2%) compared to LC (0.4–1.5%) [22]. However, according to The Swedish Registry of Gallstone Surgery and Endoscopic Retrograde Cholangiopancreatography, the skills acquired to perform LC increased throughout the years, thus the rate of BDI following OC (2.8%) was more than that after LC (1.3%) [23]. On account of the increased surgical skills in LC, Halbert et al. concluded that the overall rate of LC has declined to around 0.2% [5].

We agreed in a way with recent studies, in that 81.3% of our major BDI cases followed OC or converted cases; however, LC was responsible for 84% of minor BDIs. Overall, LC was responsible for 54.3% of all cases. We concluded that the rate of major BDI was higher in OC, as the surgical skills of OC had been in a decline in recent years as advances in the laparoscopic surgical skills had been on the rise, and that the rate of minor BDI is in a rise hence the rate of LC has increased. We suggest that more studies should carefully aim to depict the incidence, and prevalence of BDI and compare the current rate of BDI after LC, OC, and robotic cholecystectomy.

Many studies in the literature agree that the most important factor that decreases the rate of BDI is surgical skill and knowledge in the prevention of BDI. All surgeons must be oriented with a critical view of safety (CVS), respect the calot’s triangle during dissection of the calot’s triangle, and be oriented and prepared with all possible biliary tree and hepatic artery anomalies. Disregarding the CVS, the use of thermal hemostats close to the main biliary system, or strenuous traction on the cystic duct is associated with high rates of BDI [6, 7]. All surgeons should choose their patients, and the ideal timing of cholecystectomy, putting in mind the possible risks that would be encountered; for instance, performing a cholecystectomy for a patient with acute cholecystitis should be within 48 h and no more than 10 days after the onset of symptoms [9]. Therefore, the choice of the proper timing for performing a cholecystectomy is advised.

WSES guidelines recommended the use of CVS during LC and advised bailout surgeries whenever the CVS is not achievable. Bailout surgeries include Subtotal Cholecystectomy, or Cholecystostomy insertion followed by interval cholecystectomy [9, 10]. Conversion to OC is an option in challenging cholecystectomies; Although, conversion was weakly evidenced to decrease the rate of BDI [9]. We found that 28.6% of BDI cases were due to misperception of anatomy and thus the CVS was not properly identified.

Difficult cholecystectomy is a subjective term used by surgeons to indicate the difficulties encountered during cholecystectomies. Although there is no clear definition in the current literature for the term, surgeons have long used the term for the prediction of hard cholecystectomies that entail higher surgical skills [24, 25]. As such, Difficult cholecystectomy is a major cause of biliary duct injuries [24]. However, referring that most BDIs are due to a difficult cholecystectomy without objective evidence creates bias. It is not clear whether surgeons prefer to associate their complications with the fact that it was a hard cholecystectomy. It does not matter how hard a cholecystectomy is, surgeons must be able to carefully detect the CVS before ligating any structure. Respect to the CVS significantly decreases the rates of BDI. In circumstances where CVS cannot be depicted, the surgeon must undergo a “bailout” surgery [9] (we have not recorded any case that had undergone a bailout surgery, which may agree with the fact that bailout surgeries indeed avoid BDI). It is also important to note the importance of proper reporting of BDI, referring to the “ideal report” proposed by the WSES, which aims to facilitate proper identification of the hardships encountered during the procedure [9].

**Fig. 8:**
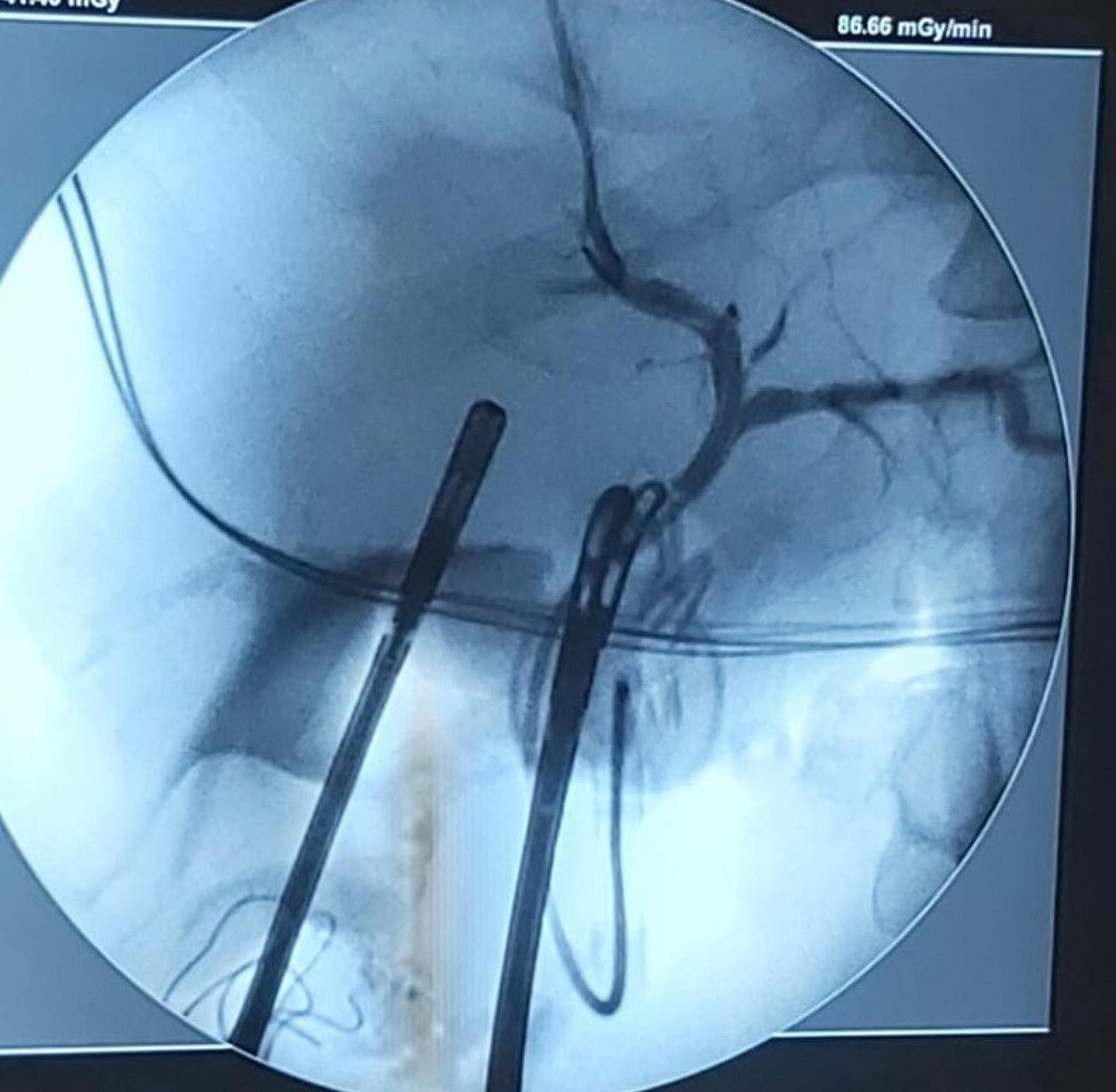
Intra-operative cholangiography with a stent seen inserted into the proximal duct

BDIs are detected in different frames of time. The earlier the timing of detection, the better the outcomes for the patient [9]. Intraoperative detection of BDI is not an easy task; however, if it occurs, surgeons must be aware of what to do next. The use of intraoperative cholangiography (IOC), and Indocyanine green-fluorescent cholangiography (ICG-C), have been proven to facilitate the detection of challenging biliary anatomy and detect BDI intraoperatively. However, there is no consensus, on the routine usage of these techniques in cholecystectomies [8, 9, 26]. A Meta-analysis undergone on 2,059 articles to evaluate the use of IOC, stated that BDI rates are lower with IOC than without IOC (depending only on the anatomical description) [27]. IOC was used in two cases in our study to help diagnose BDI (Fig. 8).

WSES recommends that once a BDI is detected intraoperatively, an experienced biliary surgeon must be consulted to assess and evaluate the condition and undertake a repair. However, if no biliary surgeon is available, it is recommended to “triage” the patient with a biliary drain and refer the patient immediately to an experienced center for further management [9].

Intraoperatively diagnosed major BDI (Strasberg E1-E5) is best definitively managed by a hepaticojejunostomy, either open or laparoscopic, if and only if, an experienced biliary surgeon is available, as this is believed to decrease the risk of post-operative leakage. Minor BDI (Strasberg A-D) diagnosed intraoperatively can be definitively managed in various ways (Fig. 2). Injury to the subhepatic bile ducts, for instance, is managed by ligation/clipping. A lateral injury without loss of tissue to a Common hepatic duct can be managed, after assessment by an IOC, by direct sutures, with or without a biliary tube (e.g. T-tube) [9].

Triaging surgeries aim to postpone the repair until an experienced biliary surgeon is available. Sadly, not all surgeons are aware of the technicality of triaging a patient diagnosed with BDI. Triaging BDI intraoperatively must only be aimed at draining the biliary system and placing a drain in the subhepatic region, then immediately referring the patient [9]. It is not justified to ligate the main biliary duct, as originally believed in old literature. Ligating the BD prolongs the hospital stay, entails more intervention to drain the bile, puts the patient at risk for cholangitis, and makes it more difficult to identify the injured bile duct during repair, due to adhesions [9].

The mainstay of treatment of Bile duct injuries is the restoration of the biliary continuity. Ligation of the BD does not treat, yet it converts one type of injury to another. Furthermore, ligation of the biliary duct disrupts the vascular supply to the retained bile duct, as the blood supply of the biliary tree is downward-upward via the ascending marginal vessels from the posterior branch of the superior pancreaticoduodenal artery [28].

28.6% (n = 11) of cases in this study were diagnosed intraoperatively, of which, 2 cases undergone primary bilioenteric repair (one laparoscopic hepaticojejunostomy, and another open hepaticojejunostomy), 7 cases were found to be minor injuries to the subhepatic ducts and were ligated, and 2 cases were triaged (one by a T-tube biliary drainage, another by ligation of CBD).

**Fig. 9:**
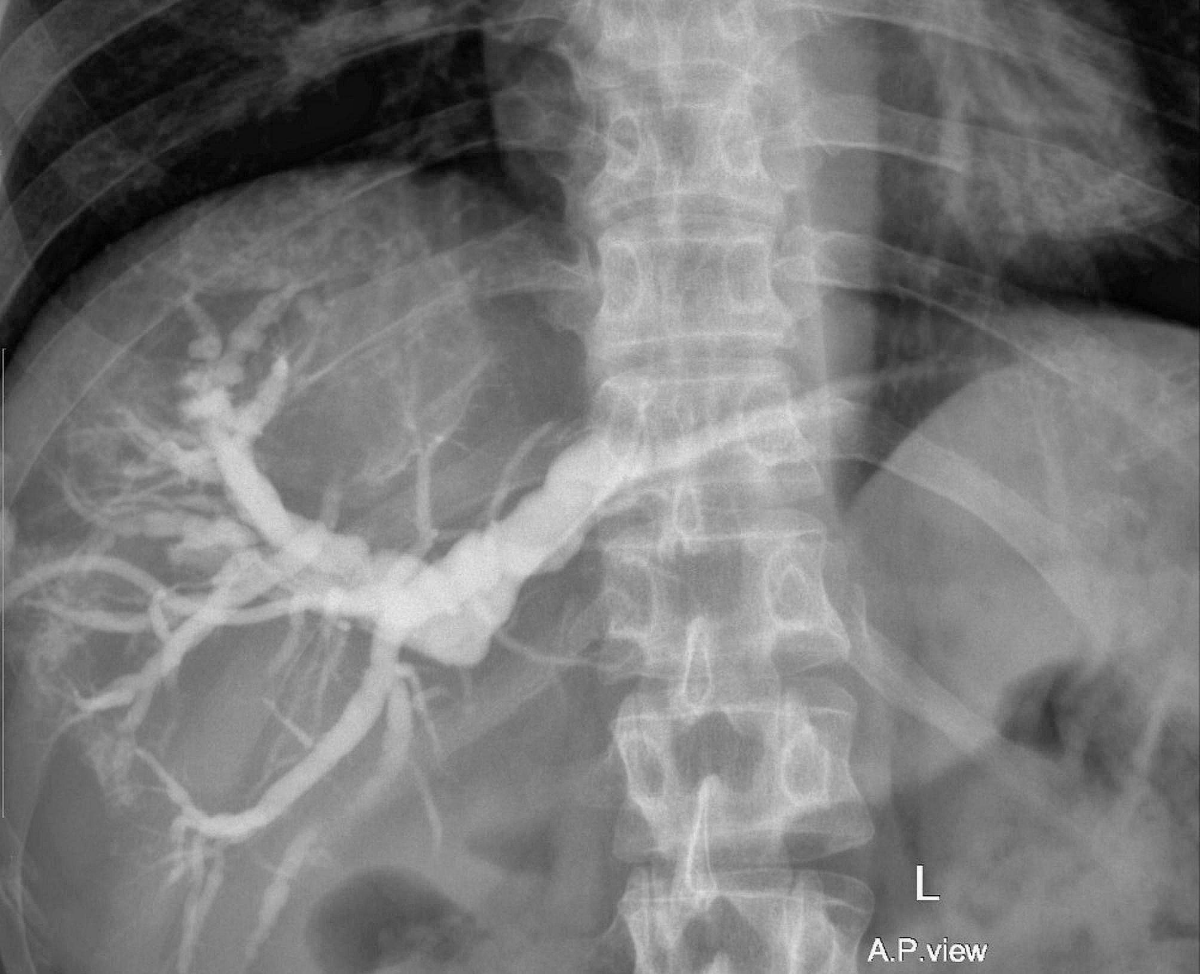
Pre-operative cholangiography of a major bile duct injury, Strasberg E2

Unfortunately, none of the triaged patients were referred immediately to our center. Ligation of the CBD had further put the patient in a prolonged management pattern, as the patient developed hyperbilirubinemia, cholangitis, fever, and abdominal pain. Furthermore, the definitive bypass was delayed long enough, to treat the sequel of such a maneuver. The patient was referred after 4 days of surgery, and PTBD was used to drain BD, to treat jaundice and cholangitis (Fig. 9). The patient was operated on for bypass after 10 weeks, after improvement of the general condition, and surgery was even harder than anticipated, due to the recurrent attacks of cholangitis the patient encountered. Thus, ligating BDs after the diagnosis of BDI intraoperatively delays rather than promotes the patient’s health and well-being.

Postoperative diagnosis of BDI is an obligatory skill for all surgeons. Symptomatology of BDI may vary from one patient to another, according to the type of injury and its extent. Subjectively, these patients often report abdominal pain, nausea, fever, and malaise. Objectively, they may show evidence of obstructive jaundice, sepsis, biliary peritonitis, or documented bile leakage from drains [29].

For patients with suspected BDI postoperatively, ultrasound remains the most used initial investigation as it diagnoses intraperitoneal collections and biliary and intrahepatic dilatation [13]. In this study, ultrasound was conducted on 89% of patients, as a primitive scanning technique. Abdominal Computed Tomography (CT) scans, on the other hand, were not conducted routinely (59.3%), except for patients with MBDI, with suspected vascular injuries, or with signs of fever, to exclude intrahepatic abscesses. CT is usually conserved for cases where Magnetic Resonance Cholangio Pancreatography (MRCP) is contraindicated, or for cases with suspected vasculobiliary injury [13].

CT scans were only conducted in 59.3% of the cases, this is because the majority of the cases (54.3%) in this study were NMBDI. The low incidence of VBI in our study might be attributed to the majority of our cases being treated with open surgery [30]. These findings align with the results of Tidjane et al. [31].

**Fig. 10:**
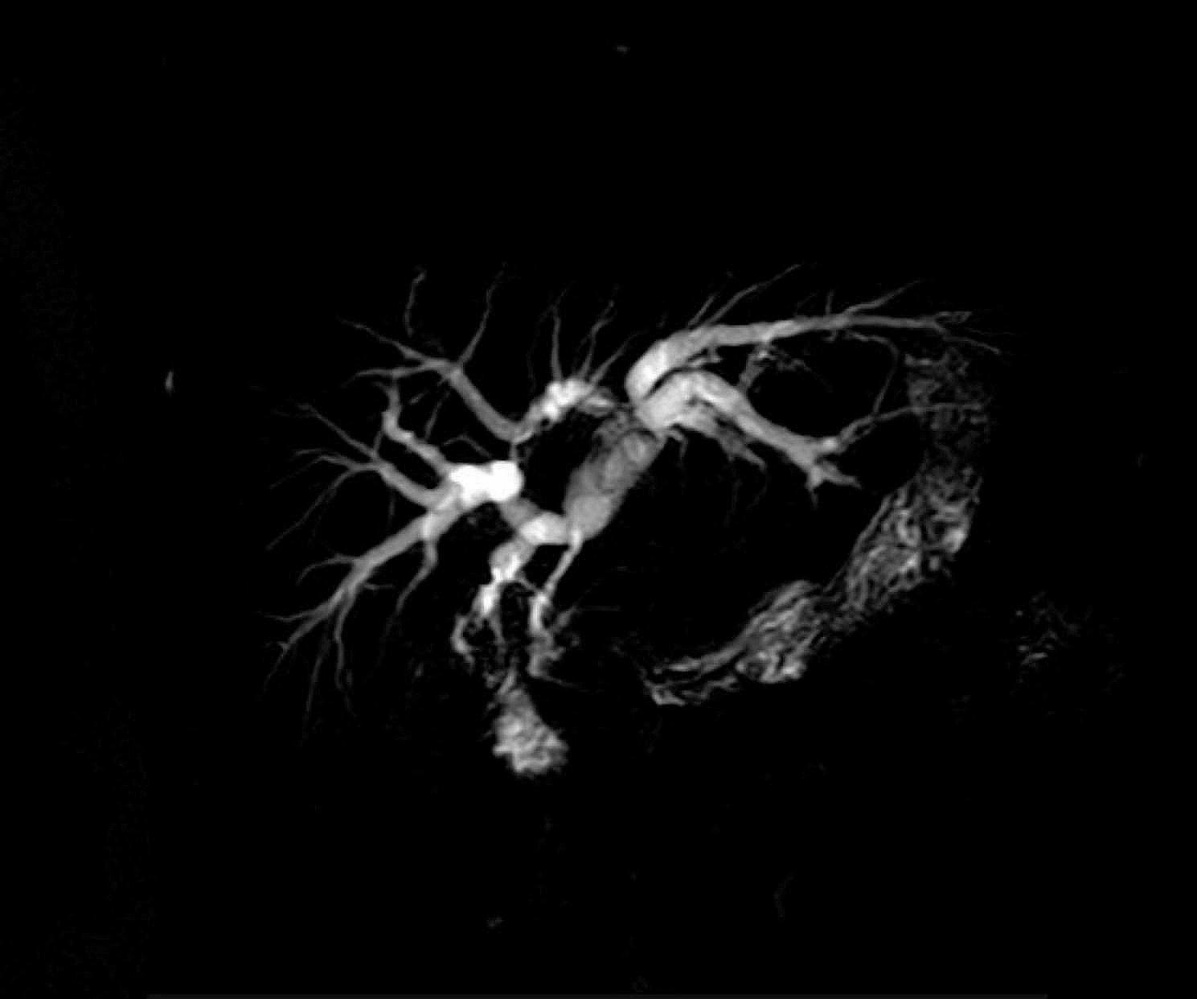
Pre-operative MRCP of a major bile duct injury, Strasberg E2

There is a general agreement toward the use of MRCP, as the gold standard, diagnostic modality in BDIs [9] (Fig. 10). 74% of our cases underwent MRCP and were classified accordingly. MRCP can be sufficient in most cases to diagnose BDI and implement the management plan.

Tube cholangiography is an efficient non-invasive modality. Tube cholangiography is cholangiography via an intraoperatively placed drain inside the biliary tree, conducted in properly triaged cases. It provides a feasible diagnostic, and follow-up modality in cases with BDI. Tube cholangiography can delineate the biliary tree, without the need for further investigations. In this study two cases had a tube cholangiography, one had it inserted as an intraoperative triaging method (T-Tube), and the other was inserted in a surgical Exploration conducted on a patient in septic shock. In both cases, tube cholangiography decreased the need for further interventions.

Invasive diagnostic methods such as Endoscopic Retrograde Cholangiography (ERCP), Percutaneous transhepatic cholangiogram (PTC), and Diagnostic Laparoscopy (DL) are not the preferred first options. Although they provide efficient therapeutic add-ons. They are usually left as second options in investigating BDIs. The literature encourages the use of ERCP, over Percutaneous Transhepatic Biliary Drain (PTBD) as its insertion can be technically difficult because intrahepatic bile ducts are usually not dilated; moreover, PTBD is advised if ERCP has failed [9]. However, our results disagree with this statement. Out of the 12 cases that have undergone ERCP, 4 cases have failed, and 1 case has died due to post-ERCP pancreatitis, indicating the high failure rate of ERCP in comparison to PTBD. Moreover, the complications of ERCP are irreversible, and moribund (pancreatitis, and duodenal perforation), in comparison to the reversible complications of PTBD (bleeding, cholangitis). PTBD is not only used as a diagnostic method to perform a cholangiogram but also a therapeutic measure to drain and stent the biliary tree and bypass partial BDI to give it a chance to heal, reserving further interventions. ERCP, however, has one advantage over PTBD, in that it has treated minor BDIs efficiently post re-laparoscopy by sphincterotomy, as a modality to decrease intra-biliary pressure. Furthermore, PTC can delineate the bile ducts more clearly and can be used in all cases, in comparison to ERCP, where discontinuity of the biliary tree, such as in cases of segment loss, hinders the performance of ERCP. Clinical trials should be conducted to compare both modalities in investigating and treating BDIs.

Relaparoscopy and diagnostic laparoscopy (DL), are two terms we used interchangeably; however, DL was used to indicate the technique used to diagnose BDI, while relaparoscopy was used to indicate the procedure to treat BDI. However, all 10 cases which underwent DL, had undergone a therapeutic intervention, which permits the usage of both terms interchangeably. Relaparoscopy has diverse uses as it allows intra-abdominal lavage, drainage of biloma, and intra-abdominal collection with the placement of a drain, clipping/ligating of any accessory ducts or an insecure cystic duct, inspection of the biliary anatomy with potential treatment (i.e., releasing a clipped CBD), performing cholangiogram, or placement of a biliary tube to facilitate tube cholangiography, which can be useful in identifying whether a persistent fistula remains in the biliary tree [16]. More trials should be undertaken to assess relaparoscopy in the management of BDI, as it seems an effective modality.

Relaparoscopy was used in 10 cases in the early postoperative period, no further intervention was obligatory; however, complementary ERCP and sphincterotomy were done to enhance the biliary drainage by decreasing the intra-biliary pressure. One case had ERCP, and a stent inserted before re-laparoscopy. The WSES recommends that cases presented in a tertiary hospital with major BDI, proceed for hepaticojejunostomy [9]. However, recent guidelines do not answer the role of re-laparoscopy, especially in the management of minor BDI, and its role in the early detection of BDI. In our study, we noticed that cases managed by re-laparoscopy in the early postoperative period avoided further sequel, in comparison to minor cases that were not managed by re-laparoscopy and were left for PTBD. Relaparoscopy was aimed not only for diagnosis, but also for lavage, drainage of the abdominal cavity, and definitively managing minor injuries.

In our study, relaparoscopy was not associated with further complications; furthermore, relaparoscopy was associated with faster diagnosis and better surgical outcomes than other modalities in the treatment of minor BDIs. The fact that the mortality rate was recorded in two minor injuries, raises the concern that minor BDIs should be studied more efficiently and that the role of relaparoscopy should be highlighted.

Managing cases with ERCP and stenting as the first modality should be evaluated in further clinical trials and studies. In our study, ERCP has been associated with a high failure rate (33.3%; n = 4) and was responsible for death in one case. On the contrary, ERCP was conducted as a first modality in one case that helped diagnose cystic stump ligature slippage and was used in its management by stent insertion; however, relaparoscopy aided in further management by applying a ligature to the cystic stump. Comparative clinical trials between ERCP and PTBD in the management of BDI should be conducted.

Classifying BDI remains the most important aspect in defining the injury, planning the management, and predicting the outcome of the proposed management. The ATOM is a thorough classification compared to all other classifications mentioned in the literature. Although it needs more knowledge, and practice to fully define a BDI using an ATOM classification, it remains a strong method that carefully depicts the type, extent, vascular association, timing of Injury, and mechanism of injury [17]. We have used both the Strasberg classification [20] and the ATOM classification in defining our cases. Our institute initially followed the Strasberg classification, but since 2020, we have followed the ATOM classification, as we observed that using the Strasberg classification did not add to defining or managing BDI. Furthermore, Strasberg’s classification did not describe VBI, timing of injury, mechanism of injury, or timing of diagnosis. Although it was harder to depict our cases using the ATOM classification, it was more apothegmatic and compact.

However, our study also highlighted that the ATOM classification was more challenging to use, especially as most of our cases (62.9%) were referred from other centers. We faced difficulties in obtaining intraoperative surgical reports, which led to an increased number of missed information regarding the mechanism of injury. Therefore, we recommend the establishment of a BDI checklist that covers all difficulties faced during the primary surgery to help report BDI accurately. The WSES recommends reporting BDI concerning the CVS scheme, mentioning any anatomical abnormality or unusual findings, such as bile drainage from a location other than the gallbladder, bile draining from a tubular structure, a second cystic artery or large artery posterior to the cystic duct, a short cystic duct, a bile duct that can be traced to the duodenum, and severe hemorrhage or inflammation. Additionally, the WSES advocates videotaping all surgeries and submitting these videos if the patient is to be referred to a secondary hospital [9].

We reviewed our cases regarding the Clavien-Dindo classification [19], to assess and evaluate management modalities and their effect on patients with morbidity and their general well-being. The classification and comparison were based on the type of therapy required to treat the complication (Supplementary File 1, Table 5). Our justification was to eliminate subjective interpretation of serious complications.

Intraoperative primary repair has many advantages over delayed primary repair in that: it is performed under the same anesthesia, avoids referring the patient to another institution, has shorter hospital stays, requires less intervention, causes less psychological trauma, and families are less likely to make malpractice litigations [32]. In our study, we noticed that intra-operative primary bilioenteric bypass was associated with lower Clavien-Dindo classes, as patients needed fewer postoperative interventions (Supplementary file 1, Table 5). hepaticojejunostomy is not confined to conventional surgeries, laparoscopic hepaticojejunostomy is a useful modality that is condoned and undertaken to treat BDIs with less hospital stay and transfusion rates [33].

In this study delayed bilioenteric anastomosis, was fashioned by Roux-en-Y hepaticojejunostomy. Hepaticojejunostomy was constructed in 48.6%, 45.7% did not need any reconstruction, and 5.7% died before definitive management. Hepaticojejunostomy is the mainstay treatment of BDIs in most scenarios [34]. Cases that did not need reconstruction were either because they were managed by other modalities such as relaparoscopy (28.6%) or were minor BDI and were managed intraoperatively (17.1%).

Leakage was recorded in 20% of the cases, and 5.7% needed further intervention after biliary leakage, in the form of Percutaneous transhepatic stenting and diversion. Both cases healed on their own, and the stent was removed 6 weeks later. Follow-up MRCP was done at 3 months, 4 months, and 6 months from the date of bilio-enteric anastomosis, with no recorded stricture up until the date of this article. Both cases were classified as grade III according to the Terblanche classification [18].

Biliary leakage post-bilioenteric anastomosis is a common complication, that can be resolved with proper drainage and medications. A retrospective cohort study in 2015 conducted on 120 cases showed a biliary leakage rate of 19.2% [35]. Management of biliary “failed” Roux-en-Y hepaticojejunostomy can entail various surgical and interventional options. Patients with failed hepaticojejunostomy may be amenable to liver transplantation [34].

It should be noted that the study has limitations due to its retrospective nature and the small sample size, which is a result of the limited number of cases referred to a single unit, and the decreased incidence of the condition. Furthermore, most of the cases reported were referred from another hospital with no surgical report, which limited the intraoperative data, especially the mechanism of injury.

## Conclusion

Managing BDI remains a concern for all surgeons, and updates in the technologies should be properly utilized in managing BDI. Surgeons must be aware of primary triaging techniques and improve their knowledge regarding triaging bail-out surgeries, both to prevent BDI and avoid the accumulation of the risk of patient morbidity and mortality in the pre-reconstructive period. The role of re-laparoscopy should gain more interest in research, as it showed preliminary value in managing BDI. Further research should focus on developing a proper checklist, that should be designed to report BDI and unify the language by which surgeons refer biliary cases.

### Electronic supplementary material

Below is the link to the electronic supplementary material.

Supplementary Material 1

Supplementary Material 2

## Data Availability

The datasets used and/or analyzed during the current study are available within the article and its additional files.

## Abbreviations

BDI: Bile Duct Injury
MBDI: Main Bile Duct Injury
NMBDI: Non-Main Bile Duct Injury
LC: Laparoscopic Cholecystectomy
OC: Open Cholecystectomy
CVS: Critical View of Safety
WSES: World Society of Emergency Surgery
CT: Computerized Tomography
MRCP: Magnetic Resonance Cholangiopancreatography
PTC: Percutaneous Transhepatic Cholangiography
ERCP: Endoscopic Retrograde Cholangiopancreatography
PTBD: Percutaneous Transhepatic Biliary Drainage
DL: Diagnostic Laparoscopy
CBD: Common Bile Duct
Ei: Early Intraoperative
Ep: Early Postoperative
L: Late
VBI: Vasculobiliary Injury
IOC: Intraoperative Cholangiography
AC: Acute Cholecystitis
