# Correction: Post-cholecystectomy bile duct injuries: a retrospective cohort study

**DOI:** 10.1186/s12893-024-02332-3

**Published:** 2024-02-07

**Authors:** Mohamed Hossam El-Din Zidan, Mostafa Seif-Eldeen, Abdelhamid A Ghazal, Mustafa Refaie

**Affiliations:** 1 Faculty of Medicine, Alexandria University, Alexandria, Egypt; 2 Alexandria Main University Hospital, Alexandria University, Alexandria, Egypt

**BMC Surgery (2024) 24:8**

10.1186/s12893-023-02301-2

Following publication of the original article [1], the caption of the Figs. 1-10 were swapped and now it has been corrected.

The original article has been corrected.
