# Supplementary material for: Post-cholecystectomy bile duct injuries: a retrospective cohort study
Source: BMC Surg. 2024 Jan 3;24:8. doi: 10.1186/s12893-023-02301-2 (PMC10765830; doi:10.1186/s12893-023-02301-2)
Supplement: Supplementary file 1 — Supplementary Material 1 [file 12893_2023_2301_MOESM1_ESM.docx]

Table 1: Distribution of the studied cases according to transferal (n=35)

| **Post-operative** | **No.** | **%** |
| --- | --- | --- |
| **Transferal** |  |  |
| Referred from another hospital | 22 | 62.9 |
| Happened and managed at AMUH | 13 | 37.1 |

Table 2: Distribution of the studied cases according to Strasberg classification (n = 35)

|  | **No.** | **%** |
| --- | --- | --- |
| **Strasberg classification** |  |  |
| **A** | 16 | 45.7 |
| **B** | 0 | 0.0 |
| **C** | 1 | 2.9 |
| **D** | 2 | 5.7 |
| **E** | 16 | 45.7 |
| E1 | 6 | 17.1 |
| E2 | 6 | 17.1 |
| E3 | 1 | 2.9 |
| E4 | 1 | 2.9 |
| E5 | 2 | 5.7 |

Table 3: Types of investigations undertaken to post-operatively diagnosed cases (n=27)

| **Types of investigations undertaken to post-operatively diagnosed cases** | **Yes** | |
| --- | --- | --- |
|  | **No.** | **%** |
| **Ultrasound (n = 27)** | 24 | 88.9 |
| **MRCP (n = 27)** | 20 | 74.1 |
| **ERCP (n = 27)** | 12 | 44.4 |
| **CT with IV (n = 27)** | 16 | 59.3 |
| **PTC/PTD (n = 27)** | 14 | 51.9 |
| **DL - Diagnostic Lap (n = 27)** | 10 | 37.0 |

Table 4: Distribution of cases according to the Clavien Dino Classification, to the type of injury (n=35)

|  | **Major/Minor** | | | |
| --- | --- | --- | --- | --- |
| **Clavien Dindo Classification** | **Major (MBDI) (n = 16)** | | **Minor (NMBDI) (n = 19)** | |
|  | **No.** | **%** | **No.** | **%** |
| I | 1 | 6.3 | 6 | 31.6 |
| IIIa | 1 | 6.3 | 0 | 0.0 |
| IIIb | 13 | 81.3 | 11 | 57.9 |
| IV | 1 | 6.3 | 0 | 0.0 |
| V | 0 | 0.0 | 2 | 10.5 |

Table 5: Comparing the Clavien-Dindo Classification to the management option undertaken (n=35), one or more management modalities may have been undertaken to the same patient.

| **Management options undertaken** | **Clavien-Dindo classification** | | | | | | | | | |
| --- | --- | --- | --- | --- | --- | --- | --- | --- | --- | --- |
|  | **I (n = 7)** | | **IIIa (n= 1)** | | **IIIb (n = 24)** | | **IV (n = 1)** | | **V (n = 2)** | |
|  | **No.** | **%** | **No.** | **%** | **No.** | **%** | **No.** | **%** | **No.** | **%** |
| **PTBD** | 0 | 0.0 | 1 | 100 | 13 | 54.2 | 0 | 0.0 | 1 | 50.0 |
| **ERCP** | **0** | **0.0** | **0** | **0.0** | **6** | **25.0** | **0** | **0.0** | **2** | **100** |
| Sphincterotomy Only | 0 | 0.0 | 0 | 0.0 | 4 | 16.7 | 0 | 0.0 | 2 | 100 |
| Sphincterotomy + Stent | 0 | 0.0 | 0 | 0.0 | 2 | 8.3 | 0 | 0.0 | 0 | 0.0 |
| **Surgical Exploration** | 0 | 0.00 | 0 | 0.0 | 0 | 0.0 | 1 | 100 | 0 | 0.0 |
| **Re-Laparoscopy** | 0 | 0.0 | 0 | 0.0 | 10 | 41.7 | 0 | 0.0 | 0 | 0.0 |
| **Primary bilioenteric anastomosis** | 1 | 14.3 | 1 | 100 | 0 | 0.0 | 0 | 0.0 | 0 | 0.0 |
| **Delayed Hepaticojejunostomy** | 0 | 0.0 | 0 | 0.0 | 14 | 58.3 | 1 | 100 | 0 | 0.0 |
| **Ligature/clipping of Duct of Luschka** | 6 | 85.7 | 0 | 0.0 | 0 | 0.0 | 0 | 0.0 | 0 | 0.0 |
| **T-tube Insertion** | 0 | 0.0 | 0 | 0.0 | 1 | 4.2 | 0 | 0.0 | 0 | 0.0 |
| **Ligature of the duct** | 0 | 0.0 | 0 | 0.0 | 1 | 4.2 | 0 | 0.0 | 0 | 0.0 |
| **Pigtail drainage** | 0 | 0.0 | 0 | 0.0 | 8 | 33.3 | 0 | 0.0 | 2 | 100 |

**Table 6:** Summary of all Sequel encountered in our study. Only 9 patients showed sequel, of which 2 died, and 7 showed one or more complications.

| **Mortality and Morbidity** | **No.** | **%** |
| --- | --- | --- |
| **Encountered complications (n = 35) (morbidity and mortality)** |  |  |
| No | 28 | 80.0 |
| Yes | 9 | 25.7 |
| **Types of complications** |  |  |
| 1. Post-reconstruction leakage | 7 | 20.0 |
| 1. Did not need any further intervention and responded to conservative treatment (minor leakage) | 5 | 14.3 |
| 1. Needed further Interventional procedures and sequential follow up. | 2 | 5.7 |
| 1. Wound Infection | 4 | 11.4 |
| 1. Death | 2 | 5.7 |
| **Causes of Mortality** |  |  |
| 1 - mortality related to delay in management |  |  |
| Intra-abdominal sepsis | 1 | 2.9 |
| 2 - mortality related to undergone investigations |  |  |
| Pancreatitis post ERCP | 1 | 2.9 |
