# Supplementary material for: Post-cholecystectomy bile duct injuries: a retrospective cohort study
Source: BMC Surg. 2024 Jan 3;24:8. doi: 10.1186/s12893-023-02301-2 (PMC10765830; doi:10.1186/s12893-023-02301-2)
Supplement: Supplementary file 2 — Supplementary Material 2 [file 12893_2023_2301_MOESM2_ESM.docx]

# Classifications used in our study.

We have provided an appendix that outlines all the classifications used in our study. This appendix is intended to assist readers in comprehending the presented data. We have included references to the original works to explain these classifications. However, the tables presented in the appendix were created by us.

## 1 – Strasberg Classification

The Strasberg classification, proposed by Dr. Steven M. Strasberg in 1995, is a widely used system for anatomically defining bile duct injuries based on location [1]. The classification categorizes bile duct injuries into different types, each representing a specific type of injury, with a similar approach necessary for their repair (Table 1). Type A injuries are managed endoscopically, Type B and C require drainage of the right liver, Type D can be primarily repaired, and Type E requires Hepaticojejunostomy. Despite its limitations, due to its simple and comprehensive description, the Strasberg classification is still the most commonly used classification for bile duct injuries [2].

Table 1: Strasberg Classification (1995) [1].

| Type | Criteria |
| --- | --- |
| A | Cystic duct leaks or leaks from small ducts in the liver bed |
| B | Occlusion of a part of the right posterior sectoral duct. |
| C | Transaction without ligation of the aberrant right hepatic ducts |
| D | Lateral injuries to major bile ducts without major tissue loss |
| E | Subdivided as per Bismuth's classification into E1 to E5 |

## 2 – ATOM Classification

The EAES ATOM classification of 2013 is based on this essence [3].

The European Association for Endoscopic Surgery (EAES) proposed an all-inclusive ATOM classification in 2013 to classify bile duct injuries (BDI) [3]. This classification system is based on anatomic (A), time of detection (To) (early or late), and mechanism of injury (M) (mechanical or thermal). The anatomic characteristics of the injury are based on the anatomic level of the biliary tree of the initial injury and concomitant vasculobiliary injury. The time of detection is categorized as early (E) or late (L). The mechanism of injury may be classified as mechanical (Me) or energy-driven (ED) injury. The ATOM classification system helps in locating the anatomical and extent of the initial injury, as well as any associated vasculobiliary injury. It divides the timing of detection into early intraoperative detection (Ei), early post-operative detection (Ep), and late Post-operative detection (L). The EAES classification system also includes the cystic aberrant and accessory (hepatic bed, subhepatic, or Luschka) ducts (Table 2).

## Anatomic characteristics of the injury

The severity of biliary injury depends on its location in the biliary tree including concomitant vasculobiliary injury. The biliary tree is divided into main and nonmain biliary ducts, where the main biliary duct includes the common biliary, common hepatic, and right and left hepatic ducts as per various classifications. Table 2 shows the levels of MBDI.

Table 2: Levels of MBDI levels according to the ATOM classification [3],

| **Level** | **Description** |
| --- | --- |
| type 1 | low main BDI >2 cm distal to the inferior border of superior hepatic confluence; |
| Type 2 | middle main BDI <2 cm distal to the inferior border of superior hepatic confluence; |
| Type 3 | high main BDI involving the superior hepatic confluence but the left-right communication is preserved, usually on the roof. |
| Type 4 | High main BDI involving the superior hepatic confluence but left–right communication is interrupted, including the E6 injury of Connor and Garden. |
| Type 5 | Left or right hepatic duct injuries without injury to the superior confluence. |
| Type 6 | Isolated segmental hepatic duct injury (right anterior or posterior sectorial). |

The non-main biliary duct (NMBD in the EAES classification) includes the cystic aberrant and accessory (hepatic bed, subhepatic, or Luschka) ducts. The type as well as the circumferential and longitudinal extent of injury depends on whether the injured bile duct was initially *occluded (O)* (ligation, clip, sealed) or *divided (D)* and leaked. In both of these, the lowercase letter *‘‘c’’* is added to stand for complete interruption (ligation, clip, sealing, or division), while a partial interruption (ligation, clip, sealing, or division) is labeled *‘‘p,’*’ followed by the percentage of the circumference involved whenever this detail is known, whether there was a loss of substance between two divisions, irrespective of whether one or both of the extremities was occluded or divided (LS; the length in centimeters, whenever known, is indicated in parentheses).

Concomitant Vasculobiliary injury (VBI) is defined as an injury to both a bile duct and a nearby vessel. Our definition also includes vascular injury that occurs alone in the index operation but results in injury, such as septic complications, stricture, or liver atrophy. When the injured (whether repaired, sealed off, or ligated) vessel is known, abbreviations can be added to VBI and included.

## Time of detection

The time of detection is classified as early (E) or late (L). Within the early detection group, a separation is made between the intraoperative (Ei) and the immediate postoperative detection groups (Ep) because the latter may be accompanied by inflammation and/or sepsis, whereas the former is usually discovered by the presence of bile in the operative field or at intraoperative cholangiogram.

## Mechanism of injury

The mechanism of injury may be classified as *mechanical (Me)* (e.g., scissors, Dormia basket stone extraction) or energy-driven (ED) (e.g., cautery or ultrasonic) injury.

## 3- Clavien-Dindo Classification

The classification system known as Clavien-Dindo is widely used in the medical field to assess the severity of complications that arise from surgical procedures [4]. The grading system categorizes complications based on the type and intensity of additional interventions required to address them. It provides a standardized language that facilitates communication and comparison of complications across different surgical specialties. This system offers several advantages to the medical community. Standardized terminology enables clear communication between surgeons, researchers, and patients about the nature and severity of complications. Consistent grading also allows for accurate comparison of complication rates across different studies and surgical centers, leading to enhanced research. Additionally, hospitals can use Clavien-Dindo grades to track performance, identify areas for improvement in surgical technique and patient care, and improve transparency and surgical outcomes worldwide.

Table 3 : Clavien-Dindo Classification.

| Grade | Description |
| --- | --- |
| 1 | Any deviation from the normal postoperative course, without the need for pharmacological treatment or surgical, endoscopic, and radiological interventions |
| 2 | Alteration of normal course Requiring pharmacological treatment, Blood transfusions, or parenteral nutrition. |
| III | IIIa: Requiring surgical, endoscopic, or radiological intervention, but not under general anesthesia  IIIb: Requiring surgical, endoscopic, or radiological intervention under general anesthesia. |
| IV | IVa: life-threatening complication ICU admission causing Single organ dysfunction.  IVb: life-threatening complication ICU admission causing Multiorgan dysfunction |
| V | Death |

## 4- Terblanche classification

Terblanche classification measures the clinical outcomes of biliary stricture after bilio-enteric repair [5]. This classification was designed to test the integrity of bilioenteric anastomosis on both high and low biliary anastomosis; however, a good clinical measurement for follow-up (Table 4)

Table 4: Terblanche classification for measuring clinical outcomes post bilioenteric anastomosis.

| Grade | Description |
| --- | --- |
| I | no biliary symptoms |
| II | transitory symptoms and no current symptoms. |
| III | Biliary symptoms requiring medical treatment. |
| IV | Recurrent biliary symptoms requiring surgical correction or related to death. |

# References:

1. Strasberg SM, Hertl M, Soper NJ: **An analysis of the problem of biliary injury during laparoscopic cholecystectomy**. *J Am Coll Surg* 1995, **180**(1):101-125.

2. de'Angelis N, Catena F, Memeo R, Coccolini F, Martinez-Perez A, Romeo OM, De Simone B, Di Saverio S, Brustia R, Rhaiem R *et al*: **2020 WSES guidelines for the detection and management of bile duct injury during cholecystectomy**. *World J Emerg Surg* 2021, **16**(1):30 **doi:** 10.1186/s13017-021-00369-w.

3. Fingerhut A, Dziri C, Garden OJ, Gouma D, Millat B, Neugebauer E, Paganini A, Targarona E: **ATOM, the all-inclusive, nominal EAES classification of bile duct injuries during cholecystectomy**. *Surg Endosc* 2013, **27**(12):4608-4619 **doi:** 10.1007/s00464-013-3081-6.

4. Clavien PA, Barkun J, de Oliveira ML, Vauthey JN, Dindo D, Schulick RD, de Santibanes E, Pekolj J, Slankamenac K, Bassi C *et al*: **The Clavien-Dindo classification of surgical complications: five-year experience**. *Ann Surg* 2009, **250**(2):187-196 **doi:** 10.1097/SLA.0b013e3181b13ca2.

5. Terblanche J, Worthley CS, Spence RA, Krige JE: **High or low hepaticojejunostomy for bile duct strictures?** *Surgery* 1990, **108**(5):828-834.
